# Nuclear export regulation of COP1 by 14-3-3σ in response to DNA damage

**DOI:** 10.1186/1476-4598-9-243

**Published:** 2010-09-15

**Authors:** Chun-Hui Su, Ruiying Zhao, Guermarie Velazquez-Torres, Jian Chen, Christopher Gully, Sai-Ching J Yeung, Mong-Hong Lee

**Affiliations:** 1 Department of Molecular and Cellular Oncology, University of Texas M. D. Anderson Cancer Center, Houston, TX 77030, USA; 2 Program in Genes & Development, University of Texas Graduate School of Biomedical Sciences at Houston, Houston, TX 77030, USA; 3 Program in Cancer Biology, University of Texas Graduate School of Biomedical Sciences at Houston, Houston, TX 77030, USA; 4 Department of General Internal Medicine, Ambulatory Treatment, and Emergency Care, University of Texas M. D. Anderson Cancer Center, Houston, TX 77030, USA; 5 Department of Endocrine Neoplasia and Hormonal Disorders, University of Texas M. D. Anderson Cancer Center, Houston, TX 77030, USA

## Abstract

Mammalian constitutive photomorphogenic 1 (COP1) is a p53 E3 ubiquitin ligase involved in regulating p53 protein level. In plants, the dynamic cytoplasm/nucleus distribution of COP1 is important for its function in terms of catalyzing the degradation of target proteins. In mammalian cells, the biological consequence of cytoplasmic distribution of COP1 is not well characterized. Here, we show that DNA damage leads to the redistribution of COP1 to the cytoplasm and that 14-3-3σ, a p53 target gene product, controls COP1 subcellular localization. Investigation of the underlying mechanism suggests that COP1 S387 phosphorylation is required for COP1 to bind 14-3-3σ. Significantly, upon DNA damage, 14-3-3σ binds to phosphorylated COP1 at S387, resulting in COP1's accumulation in the cytoplasm. Cytoplasmic COP1 localization leads to its enhanced ubiquitination. We also show that N-terminal 14-3-3σ interacts with COP1 and promotes COP1 nuclear export through its NES sequence. Further, we show that COP1 is important in causing p53 nuclear exclusion. Finally, we demonstrate that 14-3-3σ targets COP1 for nuclear export, thereby preventing COP1-mediated p53 nuclear export. Together, these results define a novel, detailed mechanism for the subcellular localization and regulation of COP1 after DNA damage and provide a mechanistic explanation for the notion that 14-3-3σ's impact on the inhibition of p53 E3 ligases is an important step for p53 stabilization after DNA damage.

## Background

Mammalian constitutive photomorphogenic 1 (COP1) is an evolutionarily conserved E3 ubiquitin ligase containing a RING-finger, a coiled-coil and WD40-repeat domains. COP1 is a crucial mediator to block photomorphogenesis in the dark through the ubiquitinated proteasomal degradation of light-induced transcription factor HY5 [1]. In mammalian cells, COP1 regulates various cellular functions, such as proliferation and survival, by facilitating the degradation of physiological substrates through ubiquitin-mediated protein degradation. The ubiquitinated targets of COP1 include stress-responsive transcription factors, p53 tumor suppressor [2], c-JUN [3-5], transducer of regulated CREB activity 2 (TORC2, a glucose metabolite regulator) [6], FOXO1 [7] and nucleosome remodeling factor MTA1 [8]. It has been shown that DNA damage leads to COP1 nuclear exclusion, [9,10] however, there is a knowledge gap about how DNA damage regulates COP1's translocation from the nucleus to the cytoplasm.

The 14-3-3 proteins are a family of evolutionarily conserved regulatory chaperone molecules involved in many diverse physiological functions, including signal transduction, stress response, apoptosis and cell cycle checkpoint regulation [10,11]. In mammals, the 14-3-3 family comprises seven isoforms: β, ε, γ, ζ, η, σ, and τ, which are widely expressed in various tissues and exert their biological functions by directly binding to phosphoproteins containing the consensus motif RX (Y/F) XpSXP or RSXpSXP. This binding alters the proteins stability and/or subcellular localization [12]. 14-3-3σ was originally characterized as a human mammary epithelial-specific marker (HME1) [13], and was later found to be an essential regulator of apoptosis, cell migration, cell cycle and DNA damage response. In contrast to the other 14-3-3 family members (β, ε, γ, ζ, η, and τ), which are able to form both homo- and heterodimers with other members, 14-3-3σ can form only homodimers [14]. This unique characteristic implies that 14-3-3σ has exclusive functions and behaviors. 14-3-3σ, but not other family members, has been found to be frequently lost or decreased in various human cancers [15] and functions as a potential tumor suppressor. 14-3-3σ negatively represses AKT-induced MDM2 activation by promoting the cytoplasmic translocation of MDM2 and triggering its degradation [10,16,17]. 14-3-3σ also directly inhibits AKT-mediated tumor progression through binding-mediated suppression of AKT kinase activity [18]. 14-3-3σ also obstructs cell cycle progression and prevents tumor cell growth by inhibiting cyclin-CDK complex activity [19]. In the DNA damage response, 14-3-3σ is known to be a p53 downstream target and may serve as a regulator to prevent oxidative and DNA-damaging stress-induced mitotic checkpoint dysfunction [20]. Although 14-3-3σ may play an important role in protecting cells from DNA damage, the detailed mechanism by which 14-3-3σ modulates the DNA damage response is not well characterized.

In this study, we examined the role of 14-3-3σ in DNA damage-mediated COP1 sub-cellular localization. We found that DNA damage induced COP1 nuclear exclusion, and that this phenomenon was abrogated in 14-3-3σ-null or -knockdown cells. Further investigation revealed that 14-3-3σ physically interacted with COP1 through an ATM phosphorylation site, which is phosphorylated in response to DNA damage. Importantly, 14-3-3σ utilizes its NES to mediate COP1 nuclear export, which leads to enhanced COP1 ubiquitination. Furthermore, 14-3-3σ was shown to not only mediate COP1 nuclear export but also repress COP1-mediated p53 nuclear export. Thus, our studies of 14-3-3σ's impact on COP1-shuttling provide mechanistic insight into COP1 localization and p53 regulation during DNA damage.

## Results

### 14-3-3σ is involved in DNA damage-induced COP1 nuclear exclusion

**Figure 1:**
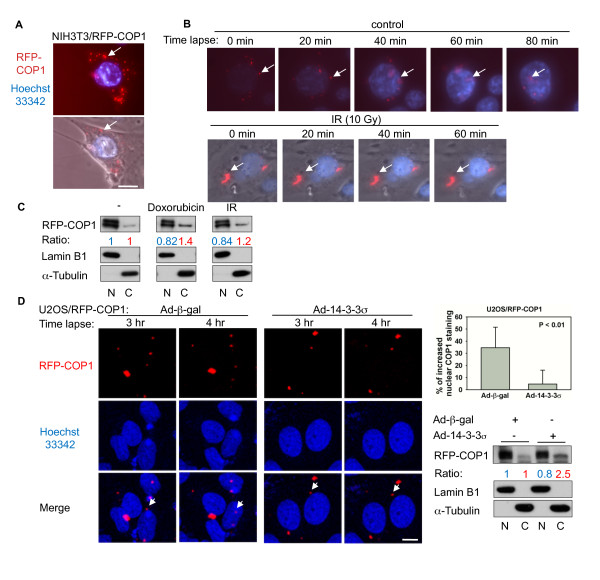
**14-3-3σ is involved in DNA damage -induced COP1 nuclear exclusion**. (A) Live-cell imaging of NIH3T3 cells expressing RFP-COP1. Typical subcellular localization of RFP-COP1 (red, indicated by arrow) is shown. Hoechst 33342 (0.04 μg/mL) was added for DNA staining (blue). Merged image containing phase-contrast image is also indicated. Scale bar, 10 μm. (B) Dynamic cell shuttling of RFP-COP1 in NIH3T3 cells was impaired in the presence of 10 Gy ionizing radiation (IR). Images were collected at 20-min intervals. The minutes indicate the elapsed time since the first picture was taken (30 min after treatment). (C) COP1 cytoplasmic localization is regulated in response to doxorubicin and IR. U2OS cells stably expressing RFP-COP1 were treated with 10 Gy ionizing radiation (IR) or 1 μg/mL doxorubicin. Subcellular localization of RFP-COP1 is shown by Western. Lamin B1 was used as the marker of nuclear (N) fraction, while α-tubulin was used as the marker for cytoplasmic (C) fraction. (D) 14-3-3σ inhibits COP1 nuclear shuttling. RFP-COP1-expressing cells were infected with Ad-β-gal or Ad-14-3-3σ. Live-cell images were captured using a confocal microscope at the indicated time points. Arrows indicate the representative COP1 translocation from the cytoplasm to the nucleus in the Ad-β-gal treatment group and lack of nuclear migration in the Ad-14-3-3σ group. Bar graph shows the percentage increase in nuclear COP1 staining in each group. Error bars represent 95% confidence intervals. Scale bar= 10 μm. Cell fractionation of RFP-COP1 in each group is shown by Western blot. (N), nuclear fraction. (C), cytoplasmic fraction.

COP1 dynamically migrates from the cytoplasm to the nucleus. This process is important to regulation of COP1's target proteins. DNA damage induces both p53 activation and COP1 subcellular shuttling from the nucleus to the cytoplasm. However, the underlying mechanism of DNA damage-induced COP1 cytoplasmic translocation is unknown. We performed live-cell imaging of NIH3T3 cells stably expressing RFP-COP1 and found punctate COP1 staining expression in both the nucleus and the cytoplasm (Figure 1A). Time-lapse confocal microscopy showed that COP1 could shuttle between the cytoplasm and the nucleus under non-stressed conditions (Figure 1B, upper panel). However, COP1's dynamic nucleus/cytoplasm shuttling was impaired in cells with DNA damage induced by ionizing radiation (IR, 10 Gy) (Figure 1B, bottom panel). We confirmed this observation in U2OS cells in the presence of IR or Doxorubicin.

Subcellular fractionation (Lamin B1 was used as the marker of nuclear (N) fraction, while α-tubulin was used as the marker for cytoplasmic fraction) followed by Western blot analysis confirmed that the proportion of COP1 in the cytoplasmic fraction increased in DNA damaged cells (treated with doxorubicin or IR) compared with untreated controls (Figure 1C). Given that 14-3-3σ can be induced by DNA damaging agents and that 14-3-3σ exerts its influence by regulating the subcellular localization of its targets [19,21], we then sought to determine whether 14-3-3σ expression can affect the subcellular location of COP1. Time-lapse confocal microscopy demonstrated that RFP-COP1 is dynamically shuttled between the nucleus and the cytoplasm in RFP-COP1-overexpressing cells infected with Ad-βgal, but this dynamic shuttling of COP1 was compromised when cells were infected with adenovirus containing14-3-3σ (Ad-14-3-3σ; Figure 1D). Subcellular fractionation followed by Western blot analysis again confirmed that the proportion of COP1 in the cytoplasmic fraction increased in Ad-14-3-3σ infected cells compared with Ad-βgal infected controls (Figure 1D). Thus, 14-3-3σ inhibited the dynamic shuttling of COP1 into the nucleus, concurrent with the increase of COP1 cytoplasmic staining.

### COP1 S387 phosphorylation is essential for 14-3-3σ-induced COP1 nuclear export

**Figure 2:**
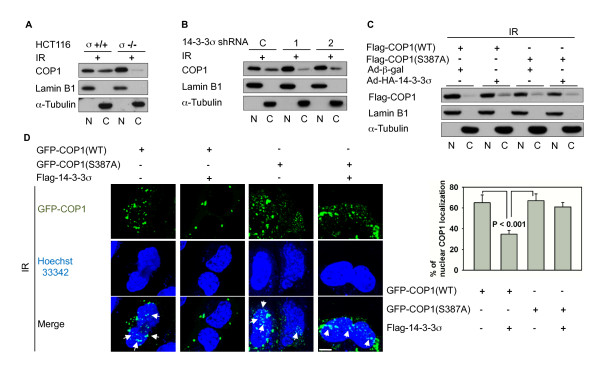
**COP1 S387 phosphorylation is essential for 14-3-3σ -induced COP1 nuclear export**. (A) Cytoplasmic COP1 localization is compromised after DNA damage in 14-3-3σ-null cells. HCT116 14-3-3σ^+/+ ^and HCT116 14-3-3σ^-/-^cells were treated with 10 Gy IR. Cytoplasmic and nuclear extracts were prepared 2 hr after irradiation. N, nuclear fraction. C, cytoplasmic fraction. (B) Cytoplasmic COP1 localization is reduced in 14-3-3σ knockdown cells after IR. HCT116 cells were stably transfected with two specific 14-3-3σ shRNA (1 & 2). Luciferase shRNA was used as a control (C). Cytoplasmic and nuclear extracts were prepared 2 hours after cells were treated with 10 Gy IR. (C) COP1 S387 mutant is less sequestered by 14-3-3σ in the cytoplasm in response to DNA damage. HEK 293 cells were transfected with Flag-COP1 (WT), or Flag-COP1 (387A), followed by infection with Ad-β-gal or Ad-14-3-3σ. Lysates were immunoblotted with anti-Flag. Cytoplasmic and nuclear extracts were prepared 2 hr after cells were treated with 10 Gy IR. (D) 14-3-3σ-induced COP1 cytoplasmic translocation is S387 phosphorylation-dependent. U2OS cells were transfected with the indicated plasmids. GFP-COP1 localization of live cell images were captured using a confocal microscope. Arrows indicate the representative location of COP1 in the nucleus. The bar graph shows the percentage of COP1 localized in the nucleus from each group. Scale bar, 10 μm.

Next, we examined the specific role of 14-3-3σ in IR-induced COP1 nuclear exclusion by irradiating both HCT116 14-3-3σ^+/+ ^cells and isogenic 14-3-3σ-null (HCT116 14-3-3σ^-/-^) cells. The loss of 14-3-3σ severely impaired IR-induced COP1 shuttling from the nucleus to the cytoplasm (Figure 2A). Knockdowns of 14-3-3σ by two distinct shRNAs also compromised IR-induced COP1 nuclear exclusion when compared with control cells infected with luciferase shRNA (Figure 2B).

It has been shown that ATM-mediated COP1 serine 387 (S387) phosphorylation leads to IR-induced COP1 nuclear exclusion [9]. To investigate whether 14-3-3σ is involved and whether facilitation of COP1 cytoplasmic translocation by 14-3-3σ is dependent on S387 phosphorylation, we constructed the S387A mutant of COP1. Figure 2C shows that the S387A mutation inhibits the increase of COP1 in the cytoplasm after Ad-14-3-3σ infection in response to DNA damage, suggesting that 14-3-3σ-mediated COP1 nuclear export is dependent on S387 phosphorylation of COP1. Next we used confocal microscopy to demonstrate that GFP-COP1 dynamic shuttling between the nucleus and the cytoplasm in cells cotransfected with 14-3-3σ is inhibited, with most GFP-COP1 accumulating in the cytoplasm (Figure 2D). On the contrary, the dynamic shuttling of GFP-COP1 (S378A) was resistant to the nuclear-export effect of 14-3-3σ expression, with most of the GFP-COP1 located in the nucleus (Figure 2D). Together, these results suggest that 14-3-3σ has an essential role in mediating IR-induced COP1 nuclear exclusion after COP1 phosphorylation at S387.

### N-terminal 14-3-3σ interacts with COP1 and promotes COP1 nuclear export

**Figure 3:**
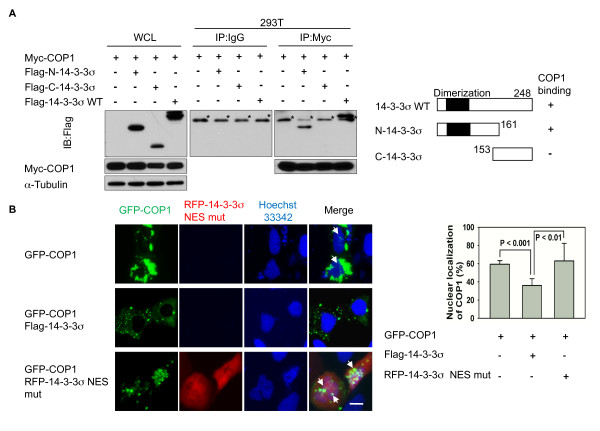
**14-3-3σ binds Cop1 and induces COP1 nuclear export via its NES**. (A) Identification of 14-3-3σ domain that interactions with COP1. 293T cells were cotransfected with Myc-COP1 and Flag-14-3-3σ wild type (WT) (aa 1-248), N-terminal (aa 1-161) or C-terminal (aa 153-248). Before harvesting, cells were treated with proteasome inhibitors. Lysates were immunoprecipitated with anti-Myc and immunoblotted with anti-Flag. Specific interactions of 14-3-3σ domains with COP1 are indicated. Asterisks indicate IgG light chain. WCL, whole cell lysate. (B) 14-3-3σ nuclear export signal (NES) mutant is unable to mediate cytoplasmic accumulation of COP1. U2OS cells were cotransfected as indicated with GFP-COP1, Flag-14-3-3σ wild type (WT), or RFP-14-3-3σ NES (I205A, L208A) mutant. Live-cell images of GFP-COP1 localization were captured using a fluorescence microscope. The bar graph shows quantification of the nuclear localization of COP1. Error bars represent 95% confidence intervals. Scale bar, 10 μm.

An issue is how 14-3-3σ regulates the nuclear export of phosphorylated COP1. We showed that there is an interaction between endogenous 14-3-3σ and COP1 by reciprocal co-immunoprecipitation (Figure 3A). To define the 14-3-3σ region responsible on 14-3-3σ required for binding to COP1, we performed co-immunoprecipitation assays using N and C terminal deletions constructs of 14-3-3σ. We showed that COP1 bound to the N-terminus of 14-3-3σ (aa 1-161) but not the C-terminus (aa 153 - 248) (Figure 3A). These data indicated that 14-3-3σ used its N-terminal region to bind to the COP1. To investigate whether 14-3-3σ associates with COP1 and utilizes 14-3-3σ's NES for mediating COP1 nuclear export, we have constructed a 14-3-3σ NES (I205A, L208A) mutant [19]. Again, we employed immunofluorescence microscopy to demonstrate that GFP-COP1's dynamic shuttling between the nucleus and the cytoplasm in GFP-COP1-overexpressing cells cotransfected with wt 14-3-3σ is inhibited by 14-3-3σ expression, with most GFP-COP1 accumulating in the cytoplasm (Figure 3B). In contrast, the nuclear export of COP1 was less affected when cells were cotransfected with RFP-14-3-3σ NES (I205A, L208A) mutant, suggesting that the nuclear export signal (NES) of 14-3-3σ is essential for the increase of COP1 in the cytoplasmic fraction. The bar graph showed that the 14-3-3σ NES mutant had less ability to reduce the nuclear accumulation of COP1 than did the14-3-3σ WT.

### 14-3-3σ is a phosphorylated COP1 binding protein

**Figure 4:**
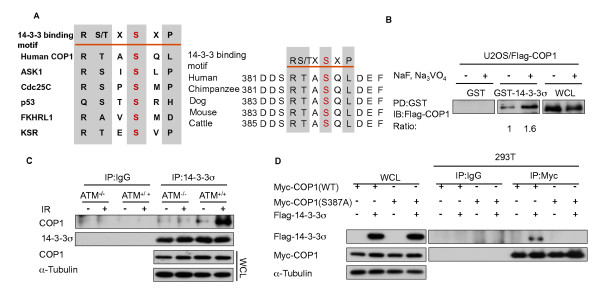
**14-3-3σ binds phosphorylated COP1**. (A) The consensus 14-3-3 binding motif is highlighted. Sequences of COP1 and other known 14-3-3 substrates are shown for comparison. Sequence alignment of COP1 containing 14-3-3 binding motifs from different species is shown in right panel. (B) Binding between 14-3-3σ and COP1 is phosphorylation-dependent. Lysates of U2OS cells stably expressing Flag-COP1 were treated with or without sodium fluoride and sodium orthovanadate. Before harvesting, cells were treated with MG132. Lysates were incubated with purified GST-14-3-3σ or GST and immunoblotted with anti-Flag. PD: pull down. (C) ATM facilitates the binding between 14-3-3σ and COP1. AT22IJE-T/pEBS7 (ATM^-/-^) and AT22IJE-T/YZ5 (ATM^+/+^) cells were treated with MG132 for 6 hrs before harvesting and were prepared at 0 (-) and 1 hr (+) after cells were treated with 10 Gy IR. Lysates were immunoprecipitated with anti-14-3-3σ and immunoblotted with anti-COP1. (D) COP1 (S387A) abolished binding to14-3-3σ *in vivo*. 293T cells were transfected with Myc-COP1 (WT [wild type]), Myc-COP1 (S387A) or Flag-14-3-3σ. Lysates were immunoprecipitated with anti-Myc and immunoblotted with anti-Flag.

14-3-3 preferentially binds to target proteins with the RSXpSXP and RXXXpSXP motifs, where pS represents phosphoserine [22]. We analyzed the COP1 peptide sequence and found that a putative 14-3-3σ binding motif [11] in COP1 is located at S387 and that this motif (RTAS^387^QL) is evolutionarily conserved in mammals (Figure 4A). We hypothesized that phosphorylation of COP1 is required for the interaction between 14-3-3σ and COP1. To address this issue, we prepared lysates from cells stably expressing Flag-COP1 with or without phosphatase inhibitors (sodium fluoride and sodium orthovanadate). The level of binding between 14-3-3σ and COP1 was lower in the absence of phosphatase inhibitors than the level of binding in their presence (Figure 4B), suggesting that phosphorylation is required for binding. To further demonstrate whether ATM-mediated phosphorylation on COP1 is important for 14-3-3σ binding, we investigated the binding between 14-3-3σ and COP1 while modulating ATM status (Figure 4C). AT22IJE-T cells [ataxia-telangiectasia mutated protein (ATM)^-/- ^fibroblasts] and AT22IJE-TpEBS7-YZ5 cells (ATM^-/- ^fibroblasts complemented with ATM cDNA) [23] were used in these experiments. We found that binding between 14-3-3σ and COP1 was increased in the presence of ATM following DNA damage but this binding was abolished in ATM deficient (ATM^-/-^) cells [23] (Figure 4C). Since S387 is an ATM phosphorylation site and the level of binding between 14-3-3σ and COP1 was higher in the presence of phosphatase inhibitors (Figure 4B), we predicted that the abolishment of COP1 S387 phosphorylation by mutating serine to alanine would interfere with 14-3-3σ-COP1 binding. Coimmunoprecipitation showed that the COP1 (S387A) mutant lost its binding affinity for 14-3-3σ (Figure 4D). These results indicate that COP1 S387 phosphorylation is required for COP1-14-3-3σ binding.

### Phosphorylation of COP1 on S387 is critical for 14-3-3σ -mediated COP1 degradation

**Figure 5:**
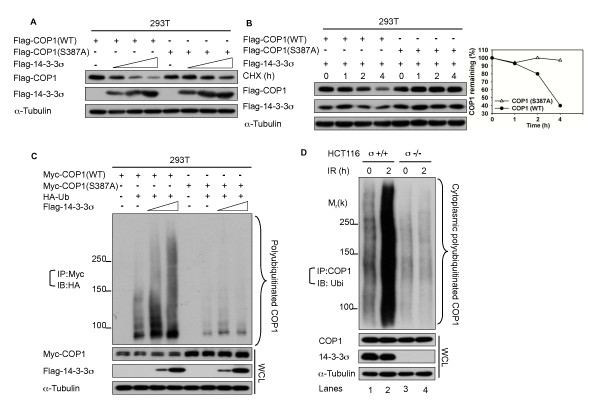
**Phosphorylation of COP1 at S387 is important for 14-3-3σ-mediated COP1 Degradation**. (A) 14-3-3σ downregulates steady-state expression of COP1 (WT) but has no impact on COP1 (S387A). 293T cells were cotransfected with the indicated Flag-COP1 (WT), Flag- COP1 (S387A) and Flag-14-3-3σ. Cell lysates were immunoblotted with anti-Flag and anti-tubulin. (B) 14-3-3σ increases turnover rate of COP1 (WT) but has no impact on COP1 (S387A).293T cells were cotransfected with indicated plasmids. Before harvesting, cells were treated with cycloheximide (CHX) for the indicated times. Cell lysates were immunoblotted with anti-Flag and anti-tubulin. Turnover rate of COP1 is indicated as a bar graph. (C) 14-3-3σ promotes COP1 (WT) polyubiquitination but has no effect on COP1 (S387A).293T cells were cotransfected as indicated with Myc-COP1 (WT), Myc-COP1 (S387A) or Flag-14-3-3σ. Cells were treated with MG132 for 6 hrs before harvesting. Ubiquitinated COP1 was immunoprecipitated with anti-Myc and immunoblotted with anti-HA. Equal amounts of whole cell lysate were immunoblotted with anti-Myc, anti-Flag or anti-tubulin. (D) 14-3-3σ promotes cytoplasmic COP1 polyubiquitination in response to DNA damage. HCT116 14-3-3σ^+/+ ^(lanes 1 and 2) and HCT116 14-3-3σ^-/- ^(lanes 3 and 4) were treated with MG132 for 6 hrs before harvesting. Cytoplasmic extracts were prepared at 0 and 2 hr after cells were treated with 10 Gy IR. Cytoplasmic polyubiquitinated COP1 was detected by immunoprecipitation with anti-COP1 and immunoblotting with anti-ubiquitin in indicated cells.

Our findings that the COP1 (S387A) mutant lost its affinity for 14-3-3σ (Figure 4D) imply that the COP1 (S387A) mutant is resistant to 14-3-3σ-induced degradation via the polyubiquitination-proteasomal pathway. Indeed, we showed that 14-3-3σ decreased the steady-state level of wild-type COP1 protein in a dose-dependent manner, while the steady state expression of COP1 (S387A) mutant is not affected by the expression of 14-3-3σ (Figure 5A). Consistently, we showed that 14-3-3σ expression had an impact on the turnover rate of COP1 but had no effect in increasing the turnover of COP1 (S387A) mutant (Figure 5B). Accordingly, the ubiquitination level of COP1 was increased by cotransfection of 14-3-3σ (Figure 5C), while COP1 (S387A) mutant was resistant to 14-3-3σ-induced COP1 polyubiquitination (Figure 5C). Given that 14-3-3σ has a role in COP1 nuclear exclusion (Figure 1) and is facilitating COP1 ubiquitination, we further addressed whether cytoplasmic COP1 ubiquitination increases upon DNA damage and whether such a process is facilitated by14-3-3σ. More cytoplasmic polyubiquitinated COP1 was detected in HCT116 14-3-3σ^+/+ ^cells treated with 10 Gy IR compared with no IR treatment (Figure 5D, lanes 1 and 2). Importantly such an increase in cytoplasmic polyubiquitinated COP1 was compromised in the absence of 14-3-3σ (Figure 5D, lanes 3 and 4). These data strongly suggest that ATM-induced COP1S387 phosphorylation is essential for 14-3-3σ-mediated COP1 degradation via the ubiquitin-proteasomal pathway.

### 14-3-3σ antagonizes COP1-mediated p53 nuclear export

**Figure 6:**
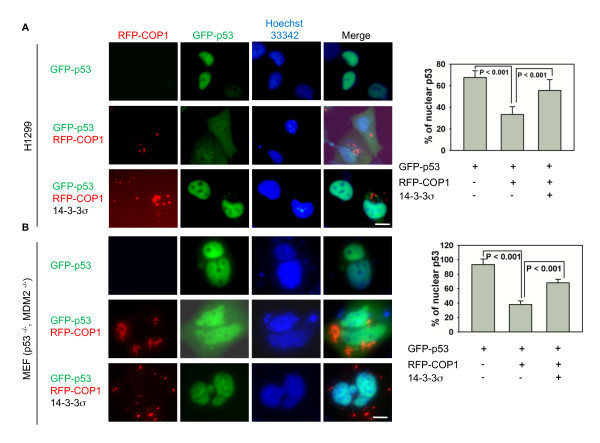
**14-3-3σ blocks COP1-mediated p53 nuclear export regardless of MDM2 status**. (A) 14-3-3σ blocks COP1-mediated p53 nuclear export. H1299 cells were cotransfected as indicated with RFP-COP1, GFP-p53 or Flag-14-3-3σ. Live-cell images were captured using a fluorescence microscope 1 day after transfection. The bar graph shows quantification of nuclear p53 localization. Error bars represent 95% confidence intervals. Scale bar, 10 μm. (B) p53^-/- ^MDM2^-/- ^MEF cells were cotransfected with the indicated plasmids. Live-cell images were captured using a fluorescence microscope 1 day after transfection as described in (A). The bar graph shows quantification of nuclear p53 localization. Error bars represent 95% confidence intervals. Scale bar, 10 μm.

It is well known that COP1 is involved in p53 ubiquitination but it remains unknown whether COP1 can regulate p53 nuclear export. To address this question, we employed immunofluoroscence microscopy and found that RFP-COP1 expression in the cell led to nuclear export of GFP-p53 (Figure 6A) in H1299 (p53 Null) cells. Given that 14-3-3σ has a role in COP1 nuclear exclusion (Figure 1) and mediates COP1 downregulation (Figure 5), we further investigated COP1-mediated p53 nuclear export in the presence of 14-3-3σ and showed that COP1-mediated p53 nuclear export is antagonized by 14-3-3σ (Figure 6A).

It has been shown that MDM2 can cause p53 nuclear export; therefore, it raises the question whether COP1-mediated p53 nuclear export is through uncharacterized activity on MDM2. To exclude the contribution of MDM2, we performed the experiments described above by cotransfecting RFP-COP1 with GFP-p53 into p53^-/- ^MDM2^-/- ^MEF cells. Our results show that RFP-COP1 expression still caused nuclear export of GFP-p53 in p53^-/- ^MDM2^-/- ^MEF cells (Figure 6B), suggesting that COP1's function is independent of MDM2. Further, COP1-mediated p53 nuclear export is again hindered by 14-3-3σ in such a context (Figure 6B). Together, these results show that 14-3-3σ's impact on COP1 nuclear export (Figure 1) is also translated into preventing COP1-mediated p53 nuclear export.

## Discussion

The dynamic cytoplasm/nucleus distribution of COP1 is important for its function. In plants, the major purpose of COP1's nuclear import is to function as a master regulator of nuclear transcription regulator HY5 [24,25], a positive regulator of photomorphogenic development. In mammalian cells, one of COP1's nuclear targets is p53, but the biological consequence of cytoplasmic distribution of COP1 is not well characterized.

Here we are able to provide important insights. First, we have shown that IR induces cytoplasmic distribution of COP1 (Figure 1) and facilitates COP1 cytoplasmic ubiquitination (Figure 5D) and that 14-3-3σ is essential for increasing DNA damageinduced cytoplasmic ubiquitination of COP1 as this activity is compromised by 14-3-3σ deficiency (Figure 5D). This 14-3-3σ-COP1 link fits very well with the notion that the inhibition of p53 E3 ligases is an important step for p53 stabilization after DNA damage. Second, we have shown that the N-terminal portion of 14-3-3σ interacts with COP1 (Figure 3) and that 14-3-3σ causes the nuclear export of COP1 through its leucine-rich nuclear export signal sequence [19], since the 14-3-3σ NES mutant loses the ability to export COP1. This observation is reminiscent of 14-3-3σ's impact on nuclear export of Cdc2 and Cdk2 [19,21]. It is important to point out that little is known about what signals or mediators control the subcellular localization of COP1. Our discovery of 14-3-3σ's role in mediating COP1 nuclear export has filled this gap in knowledge. Third, ATM-mediated phosphorylation of COP1 at S387 promotes COP1's binding to 14-3-3σ (Figure 4C). Significantly, the interaction of COP1 with 14-3-3σ is important for facilitating COP1 to stay in the cytoplasm since the COP1 (S387A) mutant, which also lost the characteristic to bind 14-3-3σ, is resistant to 14-3-3σ-mediated nuclear export (Figure 2D). Therefore, both ATM and 14-3-3σ are involved in regulating COP1 subcellular localization. Given that 14-3-3σ is also an important regulator for kinases [19,21], whether 14-3-3σ synergize with ATM to reinforce the 14-3-3σ-COP1 interaction will be another interesting layer of regulation to explore in the future. Lastly, we demonstrate for the first time that COP1 is able to cause p53 nuclear export (Figure 6), and this process is MDM2-independent (Figure 6B). Further, COP1's mediation of p53 nuclear export can still be inhibited by the expression of 14-3-3σ (Figure 6B). Because the mechanism behind COP1- mediated p53 nuclear export remains unknown, it is not clear how the nuclear COP1 binds p53 to regulate p53 nuclear export in the presence of 14-3-3σ. It is possible that 14-3-3σ binds to COP1 in the nucleus, and this binding changes the conformation and thus masks COP1's capability to export p53. Further investigation will provide the insight into this regulation. Taken together, these data suggest that the COP1 axis is independent of the MDM2 axis in terms of regulating p53 nuclear export.

It is important to point out that 14-3-3σ regulates both the MDM2-p53 axis (our previous study) [16] and the COP1-p53 axis (this study). Although the detailed mechanisms of COP1-mediated p53 nuclear export remain to be characterized, these findings highlight the complexity of the p53 nuclear export process and demonstrate that 14-3-3σ exerts negative impact on two p53 ubiquitin ligases to stabilize p53.

## Conclusion

**Figure 7:**
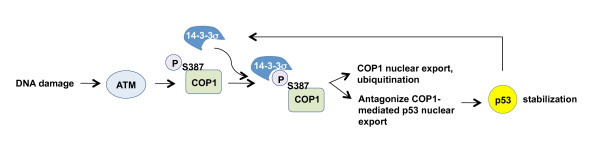
**The working model of 14-3-3σ-mediated COP1 nuclear export under DNA damage**.

On the basis of this study, we can now depict a model of COP1 regulation by 14-3-3σ (Figure 7). In unstressed cells, COP1 is mainly localized in the nucleus [9]. Upon DNA damage and ATM activation, COP1 is phosphorylated and binds to 14-3-3σ through a phosphoserine motif at S387, which is required for COP1 translocation to the cytoplasm (nuclear export). Eventually, cytoplasmic COP1 ubiquitination is enhanced, resulting in proteasomal degradation. Reducing amounts of COP1 compromises its ability to mediate p53 nuclear export, thus stabilizing p53. While ATM, 14-3-3σ, and p53 are all known to be involved in the DNA damage response, details regarding the molecular mechanism of their impacts on regulating COP1 and the biological significance of this regulation are missing. This report fills this gap in knowledge by characterizing the molecular mechanism of the ATM-14-3-3σ axis in regulating COP1 subcellular compartmentalization, and COP1 protein stability. Therefore, our research paves the path to future investigation of the 14-3- 3σ -COP1 axis as a potential therapeutic target for novel anticancer therapies.

## Materials and methods

### Cell culture and reagents

Human 293T, H1299, AT22IJE-T/pEBS7 (ATM^-/-^), AT22IJE-T/YZ5 (ATM^+/+^) and (p53^-/-^, MDM2^-/-^) MEF cells were cultured in DMEM/F12 medium supplemented with 10% fetal bovine serum. AT22IJE-T cells [ataxia-telangiectasia mutated protein (ATM)^-/- ^fibroblasts] and AT22IJE-TpEBS7-YZ5 cells (ATM^+/+^, ATM^-/- ^fibroblasts complemented with ATM cDNA) [23] were gifts from Dr. Y. Shiloh (Tel Aviv University, Tel Aviv, Israel). HCT116 and U2OS cells were maintained in McCoy's 5A medium. For transient transfection, cells were transfected with DNA using either Lipofectamine 2000 (Invitrogen), or FuGENE HD (Roche) reagents.

### Plasmids, reagents and antibodies

N-terminal Flag-14-3-3σ (1-161), and C-terminal Flag-14-3-3σ (153-248) have been previously described [17]. RFP-14-3-3σ or RFP-COP1 was cloned into pdsRed1-C1 vector. RFP-14-3-3σ (NES I205A, L208A) mutant [19], GFP-COP1 (S387A) or Flag- COP1 (S387A) was constructed by PCR cloning. Ad-14-3-3σ and Ad-β-gal viruses [20] were produced as previously described [18]. GFP-p53 was kindly provided by Dr G. Wahl [26]. GFP-MDM2 was a kind gift from Dr Sudha Shenoy. Antibodies were purchased from the indicated vendors: Flag (M2 monoclonal antibody, Sigma), tubulin (Sigma), COP1 (Bethyl Laboratories), 14-3-3σ RDI), Myc (mouse monoclonal 9E10, Santa Cruz Biotechnology; rabbit polyclonal, Sigma), HA (12CA5, Roche), His (Cell Signaling), ubiquitin (Zymed Laboratories, Inc.) and Lamin B1 (Abcam).

### Generation of stable transfectants

For generation of 14-3-3σ knockdown stable cell lines, HCT116 cells were infected with Mission lentiviral shRNA transduction particles (Sigma) containing either control shRNA, 14-3-3σ shRNA (1) (5'-CCGGC CGGGT CTTCT ACCTG AAGAT CTCGA GATCT TCAGG TAGAA GACCC GGTTT TTG) or 14-3-3σ shRNA (2) (5'-CCGGG ACGAC AAGAA GCGCA TCATT CTCGA GAATG ATGCG CTTCTT GTCGT CTTTT TG). After infection, cells were selected with 2 μg/mL puromycin for 2 weeks.

For generation of RFP-tagged-COP1 (RFP-COP1) overexpression stable transfectants, U2OS cells or NIH 3T3 cells were transfected with RFP vector or RFP-COP1 plasmids by electroporation (Amaxa). Forty-eight hours after transfection, cells were selected in a culture medium containing 500 μg/mL G418 for 4 weeks.

### Cell fractionation

Cells were lysed in lysis buffer (10 mM Tris, pH 7.6, 10 mM MgCl_2_, 1 μM DTT, 0.5% NP-40, phosphatase inhibitors and protease inhibitors), incubated on ice for 30 min, and homogenized by 20 strokes in a glass homogenizer. The homogenate was centrifuged at 4000 rpm for 5 min to sediment the nuclei. The supernatant was then centrifuged at 13,200 rpm for 10 min, and the resulting supernatant was used as the cytosolic fraction. The nuclear pellet was washed three times and resuspended in regular lysis buffer to extract nuclear proteins. The extracted material was centrifuged at 16,100 g for 20 min, and the resulting supernatant was used as the nuclear fraction.

### Live-cell imaging

RFP-COP1-expressing cells were treated with DNA damaging agents, including 1 μg/mL doxorubicin and 10 Gy IR, or infected with Ad-β-gal (multiplicity of infection [MOI] = 100) or Ad-14-3-3σ (MOI = 100). Live images of cells stably expressing RFP-COP1 were captured with an Olympus FV300 microscope or Zeiss Axiovert 200 M microscope. DNA staining was performed with 0.04 μg/mL Hoechst 33342.

### Immunoprecipitation and immunoblotting

Total cell lysates were solubilized in lysis buffer and processed as previously described [19]. Lysates were immunoprecipitated with indicated antibodies. Proteins were resolved by SDS-PAGE gels and proteins transferred to polyvinylidene difluoride membranes (Millipore). Membranes were washed and incubated with primary antibodies and peroxidase-conjugated secondary antibodies (Thermo Scientific). Chemiluminescent images of immunodetected bands were recorded on X-ray films using the enhanced chemiluminescence (ECL) system (Roche).

### Ubiquitination assay

293T cells were transiently cotransfected with indicated plasmids to detect exogenous COP1 ubiquitination. HCT116 14-3-3σ^-/-^and 14-3-3σ^+/+ ^cells were used to detect endogenous COP1 ubiquitination. Cells were treated with 5 μg/mL MG132 (Sigma) for 6 hrs before harvesting. Cells were harvested and lysed with lysis buffer described above. Ubiquitinated COP1 was immunoprecipitated with anti-Myc (9E10, Santa Cruz Biotechnology) or anti-COP1 (Bethyl Laboratories) and immunoblotted with anti-HA (Roche) or anti-ubiquitin (Zymed Laboratories).

## Competing interests

The authors declare that they have no competing interests.

## Authors' contributions

CHS carried out the molecular genetic studies, and drafted the manuscript. JC, RZ, and GVT, CG carried out the immunoassays. MHL conceived of the study, and participated in its design and coordination and SJY helped to draft the manuscript. MHL wrote the manuscript. All authors read and approved the final manuscript.
